# Stakeholder engagement and pharmaceutical pricing regulation: a qualitative inquiry

**DOI:** 10.1080/20523211.2025.2550370

**Published:** 2025-09-05

**Authors:** Ye Shing Lourdes Loh, Sharon G. M. Koh, Audrey K. L. Siah, Wing Loong Cheong, Tin Tin Su

**Affiliations:** a Department of Economics, School of Business, Monash University Malaysia, Bandar Sunway, Malaysia; b School of Pharmacy, Monash University Malaysia, Bandar Sunway, Malaysia; c Jeffrey Cheah School of Medicine and Health Sciences (JCSMHS), Monash University Malaysia, Bandar Sunway, Malaysia

**Keywords:** Public health, pharmaceutical pricing policy, external reference pricing

## Abstract

**Background:**

Medicine affordability is a critical component of a country's redistributive health policies aimed at ensuring equitable access to healthcare. This study aims to investigate key stakeholders’ perspectives on pharmaceutical pricing control in Malaysia as the country is moving towards sustainable healthcare.

**Methods:**

Semi-structured interviews (*n* = 16) were conducted with a purposive sampling of key stakeholders, which included practitioners and policymakers engaged in Malaysia’s public health policy. Data were analysed using thematic analysis guided by Walt and Gilson’s [(1994). Reforming the health sector in developing countries: The central role of policy analysis. *Health Policy and Planning*, *9*(4), 353–370. https://doi.org/10.1093/heapol/9.4.353] Health Policy Triangle framework.

**Results:**

The findings indicate a range of opinions among stakeholders, with most generally favouring the implementation of pharmaceutical pricing regulation. However, concerns have been raised about potential cost transfer, where medication expenses may be shifted to other medical services. Furthermore, there are apprehensions that price controls could adversely affect the profitability of the pharmaceutical industry and impede the development of innovative drugs. Proposed measures include the introduction of price controls and the enhancement of price transparency for specific medications used to address acute and major health issues.

**Conclusion:**

Our study contributes to the current understanding of the formation of public health policies to improve social welfare through stakeholder engagement to ensure that it reflects public needs. Malaysia is a valuable example for developing countries seeking equitable access to manage rising healthcare costs. The study is crucial for understanding country-specific experiences and stakeholders’ views on pharmaceutical pricing regulations.

## Background

Pharmaceutical pricing policy and framework have become a central issue in the literature (Babar, 2022; Babar et al., 2024; Borges Dos Santos et al., 2019) since equitable access to medicine is seen as a fundamental human right (Vogler et al., 2015). In many developing countries, high medicine prices affect accessibility to crucial treatments (Tordrup et al., 2020). The challenge facing policymakers in introducing price regulation to ensure affordability is whether their policy will be effective and reflect public interests.

Malaysia operates a two-tier healthcare system consisting of public and private healthcare, where private healthcare prices rely solely on free-market mechanisms (Ahmad et al., 2019; Hassali et al., 2015). As a result, medicine price mark-ups are several times higher (Babar et al., 2007), and patients often either rely on private insurance, corporate sector employee benefits, or have to pay out-of-pocket expenditures for medical services (Ahmad et al., 2019). Out-of-pocket expenditure is substantial as it comprises an estimated 35% of the nation’s total health expenditure (MoH, 2021a).

In 2019, the Ministry of Health (MoH) proposed a price control measure for A-category medicines, which include prescription-only drugs such as antibiotics and blood pressure medications. This initiative aimed to improve the affordability of these medications, as pharmaceutical pricing in the private sector is currently unregulated and driven by market forces (Ashraf & Ong, 2021). From January 2019 to November 2020, multiple rounds of meetings and consultations were conducted to develop this proposal (MoH, 2021b).

## The absence of pricing regulation in Malaysia

To improve medicine accessibility, the Malaysian government introduced a guideline that suggested the selling price of medicines, namely the Malaysia Medicine Price Guide (MyPriMe), also formerly known as the Consumer Price Guide (CPG). However, no legal recourse can be pursued if consumers encounter disparities between actual retail prices and those stated in the MyPriMe due to the absence of legislation (MoH, 2022). Recognising the limitations of the MyPriMe, the Malaysian government proposed the Medicine Price Mechanism (MPM) in 2019, an initiative aimed at regulating pharmaceutical prices and involving External Reference Pricing (ERP) as a main component of the policy. ERP is a pharmaceutical pricing policy that uses the prices of pharmaceutical products in various countries to establish a benchmark or reference price. To date, the proposal has not taken effect and remains under review. Table 1 reflects the medicine-related plans in Malaysia.

**Table 1.** Medicine price-related plans and policies of Malaysia.

Policy	Status	Description	Delivery agents
Malaysia Medicine Price Guide (MyPriMe)	In effect	*Proposed in 2015 Content:* 1. To provide guidelines on the retail price and medicine availability for consumers. 2. Collaboration is required between the private sector to declare their selling prices. 3. No legal action will be taken if consumers find the selling price differs from the recommended price.	Pharmaceutical Services Division
Medicine Price Mechanism (MPM)	Not in effect (proposed, under review)	*Proposed in 2019 Proposed instruments of pharmaceutical pricing policy:* 1. External Reference Pricing (ERP), and 2. Price declared by single PRHs, and 3. Price negotiations. *Content:* 1. Price controls are settled on two levels: wholesaler and final retail prices. 2. Regressive mark-up* of 10-35% to the maximum wholesale price. 3. The wholesale price of medicines will be collected 3 times a year; once the price is determined, the validity of prices lasts for 2 years. 4. The development of the public domain in declaring medicine prices. 5. Legal action would be taken against companies that did not obey the regulation.	MoH; Ministry of Domestic Trade and Consumers Affairs (MDTCA); Pharmaceutical Services Division

Source: Ministry of Health Malaysia (2021b, 2022).

The Ministry of Health selected ERP (i.e. international price comparison) to regulate pharmaceutical pricing due to several factors, including its ease of implementation (Holtorf et al., 2019) and its wide adoption in many developed countries (Ashraf & Ong, 2021). Countries that use ERP as a pricing policy use ex-factory prices or the selling cost of the medicines as a reference instead of the final selling price (Gill et al., 2019). While the ERP policy has effectively reduced medicine prices (Babar, 2022), there may be unintended consequences. For instance, the potential reduction in revenue for manufacturers leads to a loss of innovation in medicine that could have benefited future patients (Incze et al., 2022). Voehler et al. (2023) posit that the implementation of an ERP policy could decrease the probability of launching innovative drugs within nine months of regulatory approval by 73%. Hence, the suitability of an ERP policy in Malaysia is debatable in terms of its cost-effectiveness.

## The rationale and aim of the study

Previous studies on pharmaceutical pricing often assess policy outcomes upon its implementation (Babar et al., 2024). This study seeks to anticipate potential outcomes by engaging key policy stakeholders before the introduction of pharmaceutical pricing controls. Our approach highlights such policies’ anticipated benefits and challenges and provides policymakers with valuable insights to refine and optimise strategies before implementation.

In 2021, the government conducted two online public consultations to gather stakeholders’ feedback to assess the feasibility and design of the ERP policy. The online consultations involved a range of stakeholders, including patient groups (though their representation was limited), pharmaceutical companies, private hospitals, pharmacies, distributors, government agencies, businesses engaged in medical tourism, and investors. All comments made during these consultations were kept anonymous. However, most of the comments collected during the consultations were negative regarding the implementation of the ERP (Loh et al., 2023). While these consultations provided valuable feedback, there is an opportunity to broaden stakeholder representation by including patient groups and the general public (Loh et al., 2023). While online feedback is advantageous for gathering information quickly, qualitative interviews provide in-depth insights by allowing probing into the policy. Our study aims to gather key stakeholders’ views, focusing on how pharmaceutical pricing regulation could be implemented to inform policy practices.

The study seeks to bridge the gap in the current literature by answering the following research questions: (1) *What are key stakeholders’ views toward implementing pharmaceutical pricing controls?*; and (2) *What are the potential challenges of implementing pharmaceutical pricing controls and recommendations to address these challenges?*

## Methods

### Study design

Our study employed a deductive approach to qualitative descriptive analysis guided by Walt and Gilson (1994) Health Triangle framework. Adopting a deductive approach using the Health Triangle framework allowed us to focus on the *Context, Process, Content* and *Actors* in health policy for data collection and for analysing data for our study. The Health Triangle framework is suitable for the study due to its holistic approach to capturing the complexities of policy development and implementation.

**Figure 1.**
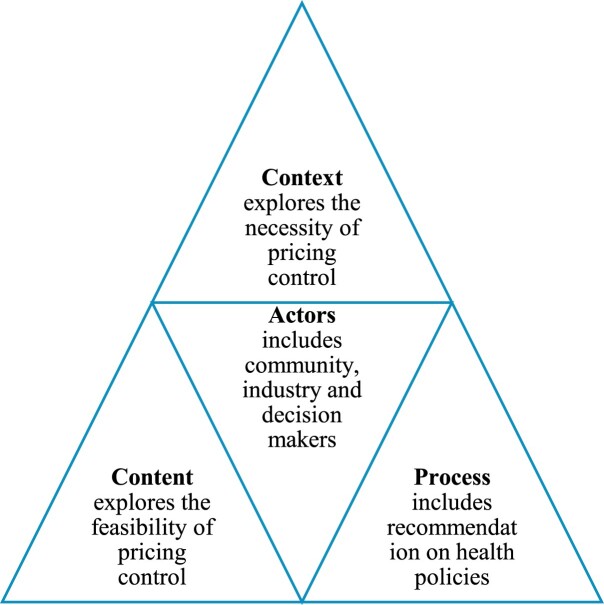
Key elements of the health policy triangle framework. Source: Author’s own illustration adapted from Walt and Gilson (1994).

As such, we applied the Health Policy Triangle framework to evaluate the prospective implementation of medicine price controls in Malaysia. Insights were gathered from key informants to deepen our understanding of potential challenges and complexities associated with implementing these price controls, as illustrated in Figure 1.

### Sampling strategy

This study used purposive sampling to recruit stakeholders involved in the policy-making of pharmaceutical pricing regulation, as identified by Loh et al. (2023). We invited pre-identified stakeholders, including healthcare professionals (such as pharmacists, general practitioners, and pharmaceutical representatives), academicians, representatives from non-governmental organisation (NGO) focused on healthcare policy, policymakers, and members of the general public. The general public was further divided into individuals with non-communicable diseases (NCDs) and those without NCDs. This assessment aims to understand the experiences of individuals who rely on long-term medication versus those who do not. It also examines the impact of price controls on medications for both groups. The participants were also recruited through snowball sampling until data saturation was achieved. Data saturation was defined as the point when no new themes or codes emerged from the data (Braun & Clarke, 2021; Hennink & Kaiser, 2022). A total of 16 key informants across all groups agreed to participate in this study (Table 2).

**Table 2.** List of participants

Stakeholders	Description	No. of participants
Pharmacists	Retailers in the pharmaceutical industry.	2
General Practitioners	Major healthcare services and consultancy providers.	2
Pharmaceutical Reps	Representative of distributors and manufacturers of pharmaceutical products.	2
Academicians	Researchers who actively conduct research related to public health policy.	2
NGO Policy Advisors	Stakeholders who had dialogues with policymakers and the government. Selected NGOs mainly focus on promoting health and social advancement.	1
Policy Makers	Governors from institutions involved in pharmaceutical pricing regulation policy decision-making.	1
General Public	Malaysian citizens who are 18 years old and above are categorised into two groups: (i) Individuals with NCDs (those who rely on long-term medication); (ii) Individuals without NCDs (those who do not rely on long-term medication).	6
		***N* = 16**

### Data collection

The interview guide was developed by referencing relevant literature focusing on stakeholder analysis of healthcare policy (Olyaaeemanesh et al., 2021; Tan et al., 2013). The interview guide was divided into two sets of questionnaires:

1. Questions for healthcare professionals, NGOs, policy advisors, pharmaceutical representatives, and the public sector;
2. Questions for the general public.

Amendments to the interview questions were made after the pilot tests to improve the clarity of the questions (see Supplemental Material A for the interview guide).

### Document review

In addition to interviews, the existing literature, mostly comprising government documents, was obtained from the websites of various government ministries. Materials related to the implementation of medicine price controls published by the relevant ministries were included in the analysis. We reviewed several policy documents (MoH, 2020, 2021b, 2022; Malaysia Productivity Corporation (MPC), 2021) to identify the key actors involved in the policy-making process.

### Data analysis

Data were analysed using qualitative thematic analysis. The Health Policy Triangle framework by Walt and Gilson (1994) was employed to organise and interpret the data based on their relevance to Context, Content, Process, and Actors (Figure 1), which are important elements of health policy. The Health Policy Triangle helps researchers and policymakers visualise the process of health policy analysis, thereby contributing to more effective implementation (Kayesa & Shung-King, 2021; Walt & Gilson, 1994).

Transcribed scripts were coded line-by-line, and codes with similar meanings were grouped into themes. The thematic analysis was done by peer debriefing with researchers of this study to derive emerging and final categories from the collected transcripts to reduce the biases of the analysis.

### Ethics approval

The study received ethics approval from the Monash University Human Research Ethics Committee (MUHREC), Project ID: 34974. All informants in this study were given an explanatory statement that included the details of the research, and verbal consent was obtained from the key informants if they agreed to participate in the study. Identifiable information will be de-identified upon transcription to prevent information breaches.

## Results

### Key issues related to **context** of pharmaceutical pricing control

Walt and Gilson (1994) assert that the context of health policy analysis extends beyond policymakers and their role in economic policy reforms to encompass the broader macro and cultural environment. Pharmaceutical pricing control is proposed to improve drug affordability by regulating medicine prices. Nonetheless, key informants from the healthcare industry highlighted that pharmaceutical companies maintain control over medicine prices through market exclusivity, whereby they limit competition and maintain high prices through patented drugs.

Healthcare providers, such as private clinics, do not have much influence in deciding the selling price of a medicine. Furthermore, there are no incentives for pharmaceutical companies to lower their selling prices, given the relatively inelastic demand for medicines, as reflected in the following excerpt,

no matter how expensive it is, there [are] still people who [are] willing to pay for it. (Pharmaceutical Representative, #1)

When asked about their views on the current medicine pricing system, a majority offered varying viewpoints on the reasons for the high medication cost, indicating a lack of transparency regarding the price determination process.

A report released by the MoH highlights another context-related factor attributing high medicine prices to price discrimination by pharmaceutical companies, where the public and private healthcare sectors are charged different prices (MoH, 2022). The statement is further validated by the representatives from the pharmaceutical industry (Table 3, pharmaceutical representative #2).

**Table 3.** Summary of key themes and informant quotes.

Elements	Themes	Category	Key Informant Views	Quotes
Key Issues Related to Context of Pharmaceutical Pricing Control	The necessity for price controls	The pharmaceutical industry determines prices	Key informants from the healthcare industry (General Practitioner, Pharmaceutical representative) mentioned that pharmaceutical companies have higher market power due to (i) the relatively inelastic demand for medicines, (ii) the pharmaceutical industry practising price discrimination, (iii) the Ambiguity of the cost of drug development.	‘Yes, for my company, the difference is not that big, but it's also about 100 ringgit difference. Because the best selling … [which is] the cheaper version, we were selling in the private sector 100+ (RM) but to the government is 20 to 30 (RM).' (Pharmaceutical Representative #1) ‘From [the] industry point of view, as a person who has worked in the industry … the pricing regime is usually [how] the products are being priced at a very high margin … most of it is around minimum 60% margin.' (Pharmaceutical Representative #2)6
		Limited influence of consumers in terms of pricing.	Key informants from the general public stated the passivity of consumers when making decisions on purchasing medicines. This is mainly due to information asymmetry, as consumers do not have sufficient information about the price of medicines.	‘I think one or two hundred (RM) is inevitable. It's not like you can negotiate the price; there's no room for bargaining. … As a consumer, you can't haggle; it's not like buying vegetables at the market where you can negotiate.' (General Public, without NCD #1) ‘Patients sometimes also have a situation that they really depend on the doctors to prescribe, due to the asymmetry of information, this kind of reliance actually bringing them disadvantage, because they probably don't know, there's also another source of generic because the doctor could be under the instruct [from] the industry to prescribe the originator.' (NGO #1)
Key Issues Related to Content of Pharmaceutical Pricing Control	Social Feasibility of Pharmaceutical Pricing Control	Differing views from informants.	Key informants have agreed that medicines provided by the public healthcare institution are affordable. Price controls should be applied to drugs treating acute and major health issues like cardiovascular diseases and asthma. Healthcare professionals (academicians, pharmacists) have pointed out the increasing costs for the government to maintain public healthcare. Therefore, ERP policy should help reduce the burden on government.	‘Seeking medical treatment at public hospitals is always affordable, even for more severe illnesses or chronic diseases, like my mother has received significant help from the public hospital' (General Public, without NCD #2) ‘Perhaps there can be control measures for certain medications, such as those for heart conditions or asthma, which are more acute or major health issues. In certain countries where the prices of some medications fluctuate significantly, there is a need for a regulation to exorbitant pricing.' (General Public, without NCD #2)
		Concerns of implementation	The general public (without NCDs) has expressed their fear of cost transfer (where the cost of medicine may transfer to other medical services) if ERP has been implemented. Policymakers pointed out that one of the possibilities of having price controls may impact the pharmaceutical industry's profitability and that innovative drugs will be compromised.	‘This policy is not very feasible because there will still be much controversy. It only comforts those in the pharmaceutical industry but does not appease others, particularly the end-users or consumers. Ultimately, the costs are still passed on to consumers, who will have to bear those expenses.' (General Public, without NCD #1)
	Operational Feasibility of Pharmaceutical Pricing Control	Institutional limitations	General practitioners and policymakers expressed the current challenges of the government: (i) IP-related issues that further burden the government with the of procuring medicines; (ii) To balance between the demands of businesses and the well-being of the public	‘Because of IP issues from the US, they (medicines) can be very expensive to procure, and there are probably limited amounts that MoH is able to procure. Even if they can procure, the price may be too expensive for the ordinary person to access.' (Policymaker #1) ‘My experience on this front is, you know, largely from an industry standpoint, and of course, the pharmaceutical industry did lobby MITI very heavily when the Ministry of Health used compulsory licensing.' (Policymaker #1)
		Constraints on public hospital	The general public mentioned the limitations of public hospitals, which include: (i)Lack of accessibility of public hospitals in remote areas; (ii) Limited capacity of drug procurement.	‘In remote and underdeveloped areas, pharmacies might sell medications at higher prices if no regulations exist. As a result, [people] in these less developed regions may have to buy medications at inflated prices, which is a negative situation.' (General Public, without NCD #2) ‘When you find medications are very expensive, you might opt to go to a public hospital. However, even at public hospitals, you have to wait for a long time, and sometimes, you can't get the medications immediately. I received my medication in March, and they informed me that I can only get it again in June, but I might have finished it by April or May.’ (General Public, with NCD #1)
Key Issues Related to Process of Implementing Pharmaceutical Pricing Control	Recommendations on government policies	Information Dissemination	Due to information asymmetry, the policy and knowledge are not well disseminated to the general public and healthcare professionals (pharmacists and general practitioners), as they hold differing views on the issue of the pricing of drugs. • NGO policymakers discussed that the government's role in public health policy-making should improve stakeholder engagement. • Pharmacists advised that the option of having generic drugs should be provided to consumers.	‘Because not just us to approach them (the government) will also be approached by the pharmaceutical [companies] or business … If (the government) doesn't know the issue enough, it will be swayed.’ (NGO #1) ‘That one (originator drugs) might be more pricier compared to the generic medicine. I think the public needs to be educated and given an option for them to choose.' (General Practitioner #3)
		Comprehensive healthcare policy (re)evaluation	Key stakeholders who were healthcare professionals (pharmaceutical representative, pharmacist and academician) suggested several evaluations of the healthcare industrial policy: (i) Implement a tiering system; (ii) Encourage mass production of generics in the long run.	‘A tiering system would mean that … for the item that is still on patent and that is new, maybe there should be a lower ceiling, versus the one that is matured, that one should be cheaper … and off-patent [drugs], of course, definitely need to be cheaper. Do not expect consumers to pay [the] full patent price.' (Pharmaceutical Representative #2) ‘If you want to improve in terms of pricing, [what] you can do from the manufacturer, you can [get] more people to involved in mass manufacturing of the medication in our own (country). If your ultimate goal is to bring down the price, that (mass production) will be the best, and you don't have to import.' (Pharmacist #2)
		Pricing regulation governance	Academicians and policymakers have commented on several policy plans to reduce the price of medicines and implement ERP policy. Academicians suggested enhancing price transparency in pharmaceutical products, and ERP policy should cater to underprivileged groups. Effective communication (negotiation) with pharmaceutical companies could be essential in achieving a consensus on medicine pricing.	‘Yeah, it needs to be controlled, to cater to all needs of Malaysians. Moreover, to have consistent pricing across all pharmacies, there should not be huge variabilities (in prices). This also depends on the state as well. Some states have different prices, and especially in urbanised areas such as KL (Kuala Lumpur), [they have] different prices. Hence, keeping it (medicine prices) consistent is good.' (Academician #2) ‘Balance the different needs of industry, the government and our patients will [need] to have some council or a [regulatory] body that can sit together, try to advise the government on what are the more sustainable policies with regard to medicine prices [and] moving forward.' (Policymaker #1)
Who are the Main Actors in policy development for Pharmaceutical Pricing Control?		Community Groups	General Public	
		Key players in the healthcare industry	Association of Private Hospitals, Malaysia (APHM), the Pharmaceutical Association of Malaysia (PhAMA), the Malaysia Medical Association (MMA), and the Malaysian Organisation of Pharmaceutical Industries (MOPI).	
		Decision-makers	Ministry of Health (MoH), Ministry of International Trade and Industry (MITI), Ministry of Finance, MyIPO, Malaysia Health Travel Council (MHTC), Malaysia Productivity Corporation (MPC), and the Central Bank of Malaysia.	

In the broader macro context, consumers are involuntarily affected by rising medicine prices. Some consumers are left vulnerable as they must accept the predetermined prices. Another challenge faced by consumers is information asymmetry and their lack of knowledge about originator drugs and generic drugs. Key informants from NGOs also noted consumers’ reliance on medical doctors to prescribe their medications for treatment, placing them at a disadvantage (Table 3, NGO #1).

### Key issues related to the **content** of pharmaceutical pricing control

Key informants in this study have pointed out several issues and goals that the proposed price control policy should address. The content of the policy needs to be adaptable and flexible enough to accommodate the current challenges of the Malaysian healthcare system. We have divided the stakeholders’ feedback into two primary categories, namely (i) Social feasibility; and (ii) Operational feasibility of implementing pharmaceutical pricing control.

### Social feasibility of pharmaceutical pricing control

The perceptions of the implementation of price controls vary among key informants. The interview quotes reflected the diverse views of key informants.

Despite the two-tier healthcare system of Malaysia, the MoH acts as the largest healthcare provider that controls and procures medicines for the public sector (Hassali et al., 2015). This explains why the public sector medicines cost less than the private sector. The view was reflected in key informants’ quotes (Table 3, general public, without NCD #2), as some respondents stated that medications in the public sector are considered affordable. This is largely due to significant subsidies on medicines in Malaysia as well as the Generic Medicines Policy, introduced in 2007, which promotes the use of generic drugs (Hassali et al., 2015). According to the respondents, price controls should be applied to drugs used to treat acute and major health issues, such as cardiovascular diseases and asthma (Table 3, general public, without NCD #2).

Several key informants have expressed concern that implementing medicine price controls may have unintended consequences, such as the cost transfer from medicine prices to other healthcare-related services (Table 3, the general public, without NCD #1). This could occur if medicine costs are capped at low levels, as healthcare providers such as private medical practitioners may offset the loss of profit by increasing the prices of health services, ultimately resulting in consumers paying higher prices for medical care. Evidence from China's nationwide pricing reform for drugs and medical services has confirmed that while the reform has effectively reduced drug prices, physicians are increasingly prescribing more examinations and tests. This trend appears to be a response to supplier-induced demand, as doctors seek to compensate for profit losses in drug sales (Zhang et al., 2020).

In addition, implementing medicine price controls may worsen medicine shortages for certain drugs as market intervention will lead to shortages, affect industry profitability, and lead to pharmaceutical company withdrawals (Acosta et al., 2019). One of the key informants (policymakers) has pointed out that the pharmaceutical industry:

may decide to pull these drugs out of the market and not allow them to be sold in Malaysia.(policymaker #1)

### Operational feasibility of pharmaceutical pricing control

Key informants from policymakers and the healthcare industry have pointed out several limitations faced by the government and pharmaceutical industry when developing pricing policies. One major consideration is the issue of intellectual property contributing to higher medicine prices and places an additional burden on the government in terms of procuring medicines (Table 3, policymaker #1). A systematic review has confirmed that intellectual property rights are typically linked to higher drug prices, resulting in increased costs for both consumers and governments in countries like Canada, India, Thailand, and Korea (Tenni et al., 2022). A general practitioner also mentioned that,

there is not much KKM (Ministry of Health Malaysia) can do without just absorbing the cost. (General Practitioner #1)

Pharmaceutical companies are actively lobbying to allow the production of generic hepatitis C drugs, which are typically cheaper than patented versions. While compulsory licensing could effectively reduce local drug prices, its implementation remains a subject of debate (Urias & Ramani, 2020) without violating intellectual property rights. Policymakers need to balance the demands of businesses as well as societal well-being.

One key informant explained that for some rural households, the inconvenience of remote healthcare facilities would leave them with no choice but to purchase medicine at much higher prices (Table 3, the general public, without NCD #2). Ahmad et al. (2019) confirmed that the price disparity of medicines between urban and rural areas contributes to market segmentation, which may have reduced the affordability of medicines.

Due to the limited capacity of drug procurement for public hospitals, some members of the general public, especially those who rely on long-term medication, experience long waiting times to get essential medication from public hospitals (Table 3, the general public, with NCD #1). Based on the challenges faced by public healthcare, a price control policy could be considered a measure to ease the government's operational burden and address high drug prices in remote areas (Table 3, general public, without NCD #1).

### Key issues related to **process** of implementing pharmaceutical pricing control

*Information Dissemination:* Key informants from the NGO discussed that policymakers’ lack of understanding of healthcare issues may have caused the policy discussion to be ineffective, as policymakers would be approached by different interest groups (Table 3, NGO #1). Croke et al. (2019) discussed this issue in the Malaysian context, where the researchers observed that these interest groups have successfully rallied public opinion against proposed reforms, which has caused political leaders to view these interest groups as having considerable public backing. In addition, key informants also stressed that

Some of the healthcare professionals’ opinions are like, very unlikely be approved, [or] to be measured. (Pharmaceutical Representative #2)

This situation is commonly identified in policy-making due to mutual mistrust and the absence of personal contact between researchers and policymakers (Oliver et al., 2014).

Apart from these institutional challenges, disseminating information on the policy content also plays a crucial part in enhancing public engagement. Key informants pointed out that:

The government, although they come from [a] public interest perspective, they did not do well in terms of disseminating that information down to the ground. (NGO #1)

*Comprehensive healthcare policy (re)evaluation:* Implementing medicine price controls requires a comprehensive assessment of the Malaysian healthcare system. The challenges faced by policymakers were compounded by the argument from the pharmaceutical industry on the loss of profit due to price controls and high R&D costs for innovative drugs. Key informants from the pharmaceutical industry emphasised that:

it shouldn’t be blanket, but there should be a tiering system. (Pharmaceutical Representative #2)

One of the common feedbacks from the key informants on healthcare policy evaluation is to promote the use of generics as a short-term strategy i.e. generic medicine policy. The availability and use of generic medicines could increase the market competition of medicines and improve their accessibility with lower prices (Tan et al., 2013). As a long-term strategy, the government should encourage mass production of generic medicines to reduce healthcare costs and encourage competition in the pharmaceutical industry (Table 3, pharmacist #2).

*Pricing Regulation Governance:* The proposed price control policy should enhance price transparency (Table 3, quoted by Academician #2). Factors affecting price transparency's feasibility are medicine price disparity between urban and rural areas (Ahmad et al., 2019) and the lack of government monitoring regarding inconsistencies in medicine pricing (Ahmad et al., 2019, 2020).

Key informants emphasised the importance of communication between key stakeholders in achieving a consensus on medicine pricing. To balance the diverse needs of healthcare providers and the general public, key informants from the public policy sector advised establishing a Council (Table 3, policy maker #1). The Council would serve the dual purpose of communicating the policy plan and acting as a bridge for discussions regarding the pricing of medicines.

### Who are the main **actors** in policy development for pharmaceutical pricing control?

The review of the policy documents (MoH, 2020, 2021b, 2022; Malaysia Productivity Corporation (MPC), 2021), identified the actors of pharmaceutical pricing regulation as the stakeholders involved in the policy development, i.e. Community groups, key players in the healthcare industry and decision-makers.

- *Community groups*: In the National Healthcare System, the general public is the targeted policy group. In past meetings and online consultations, only two patient advocacy groups were involved in policymakers’ discussions. A content analysis that analyses the public comments about implementing medicine price control reveals that the general public's voice may be lacking from past meetings (Loh et al., 2023).
- *Key players in the healthcare industry*: The healthcare industry consists of a group of healthcare providers, such as pharmaceutical companies (i.e. branded and generic manufacturers), hospitals (i.e. private and public), medical practitioners, and pharmacists (Wong et al., 2014). In past meetings, the government has been able to reach out to key players in the healthcare industry comprising organisations such as the Association of Private Hospitals, Malaysia (APHM), the Pharmaceutical Association of Malaysia (PhAMA), the Malaysia Medical Association (MMA), and The Malaysian Organisation of Pharmaceutical Industries (MOPI).
- *Decision-makers*: Key decision-makers in pharmaceutical pricing regulation, which are the government agencies, were well-represented in the decision-making process. The government and policymakers are responsible for implementing and supporting the delivery of pharmaceutical pricing regulation in Malaysia. Previous meetings on the pharmaceutical pricing regulation encompass entities such as the MoH, Ministry of Investment, Trade and Industry (MITI), Ministry of Finance (MoF), Intellectual Property Corporation of Malaysia (MyIPO), Malaysia Health Travel Council (MHTC), Malaysia Productivity Commission (MPC), and the Central Bank of Malaysia (BNM). Most of the key informants in this study have stated that the government entity with the highest authority is the MoH.

## Discussion

In this study, we analysed the key stakeholder perspectives on the introduction of ERP by applying the Walt & Gilson Health Policy Triangle framework to evaluate the potential implementation of medicine price controls in Malaysia. To address the gap from the previous study, this study highlights the significance of having clear challenges outlined and the need for a transparent healthcare system to enforce regulations on pharmaceutical pricing. Our study presents a novel approach to evaluating the implementation of medicine price controls. It distinguishes itself by focusing on evaluating stakeholders’ views prospectively rather than retrospectively assessing policy implementation. Most studies that analyse stakeholders’ views predominantly analyse the aftermath of policy implementation (Komakech et al., 2024; Mukanu et al., 2017), whereas our study aims to understand the potential outcomes by interviewing key policy stakeholders before such policies take effect.

The price control measure should serve the purpose of stabilising drug prices, which is prevalent in the proposed plan by the Malaysian government. The ERP proposed by the Malaysian Government compares the prices of medicines from reference countries, which ensures consumers do not pay more than other comparable options (WHO, 2020). Although there are various pricing models, such as value-based pricing and managed entry agreements, the successful implementation of pharmaceutical pricing regulations is highly dependent on the pharmaceutical situation of the country (Babar, 2024). For example, value-based pricing sets the prices of pharmaceutical products according to the perceived value to patients. This approach necessitates a customised pricing strategy that considers clinical value and requires strong health technology assessment capabilities (WHO, 2020). Meanwhile, managed entry agreements are contractual arrangements between pharmaceutical manufacturers and payers (Dabbous et al., 2020), which demand effective governance and negotiation skills. Considering the constraints in the Malaysian context, as indicated by the study results, ERP could serve as a practical approach for Malaysia, although other models may be explored in the future.

Notably, our findings show significant differences in stakeholder perspectives that may affect policy feasibility. For instance, while the general public is supportive of the implementation of ERP policy, private sector stakeholders, particularly from the pharmaceutical industry, expressed strong reservations as they worried price controls would hinder the research and development of innovative drugs (Loh et al., 2023). These stakeholder dynamics have posed challenges for policymakers, as the government is often lobbied by the pharmaceutical industry (refer to Table 3, quote from policymaker #1). While price controls, or ERP, may appear practical due to public support, their effectiveness in practice heavily depends on thoughtful stakeholder engagement and balancing conflicting interests. This will be discussed further in relation to pharmaceutical pricing policy.

## Implications for pharmaceutical pricing policy

Following a conceptual framework proposed by Babar (2024), effective medicine pricing policies for low and middle-income countries can be developed under: (1) data and statistics on the pharmaceutical situation of the country; (2) Having a national medicine policy in the country; (3) The availability of the medicine pricing data (4) Human resources and technical capacity. Our study aligns with the framework as we emphasised the systemic issues of the Malaysian healthcare system prior to implementing pharmaceutical pricing regulation. We will focus on discussing the main enablers along with the findings of our study.

### The pharmaceutical situation of the country and the availability of medicines data

To create effective pricing policies, it is essential to have accurate pricing information, as noted by Babar (2024). One of the main goals of implementing an ERP system is to achieve cost containment. Evidence from Turkey shows that pharmaceutical spending as a percentage of healthcare costs decreased from 36% in 2004 to 27% in 2011 (Kanavos et al., 2020). However, a major drawback in Greece's ERP implementation has led to an increase in pharmaceutical spending due to the lack of a transparent medicine price database and inappropriate calculation of medicine prices (Rémuzat et al., 2015). This could serve as a valuable lesson for Malaysia to achieve drug cost containment.

Healthcare policies such as pharmaceutical pricing control require a comprehensive policy design that aligns with the social and administrative context of the country. As discussed in the context of pharmaceutical pricing control, the ambiguity of the cost of drug development should be addressed through improving price transparency before introducing pricing regulations. Evidence from European countries such as Denmark and Norway have shown that price transparency initiatives have effectively reduced drug expenditures and prices (Brekke et al., 2011; Kaiser et al., 2014). Key informants in our study suggested that price transparency could enhance the effectiveness of the pharmaceutical pricing policy using the ERP approach by making medicine price information widely available. The Malaysian government has made efforts to create a price transparency mechanism for the local pharmaceutical industry by displaying medicine prices starting in May 2025 (Malay Mail, 2025). Despite the significant opposition from healthcare professionals, who argued that the law was improperly applied (Hassan, 2025), the implementation status remains uncertain.

To improve the availability of information on medicine prices, Gill et al. (2019) underline the need to strike a balance between health goals and industrial policy goals by incorporating input from all stakeholders. This underscores the need for the government to prioritise the establishment of a robust price transparency mechanism to ensure the success of pharmaceutical pricing policies while balancing these objectives.

### Having a (comprehensive) national medicine policy in the country

As discussed earlier in the discussion section, the challenges of balancing the interests of profit-driven pharmaceutical companies and welfare-focused governments present challenges for policymakers in enforcing drug price transparency, as identified in the main findings. To address this, policymakers could establish rules and regulations to facilitate effective negotiations with the pharmaceutical industry: a practice common in the United Kingdom, France and Germany prior to the implementation of pharmaceutical pricing controls. This includes establishing a pricing committee to facilitate negotiations between pharmaceutical companies and the government (Rodwin, 2021). One of the successful examples in Southeast Asia is Singapore’s Agency for Care Effectiveness (ACE), established in 2015, which has significantly evaluated and negotiated drug prices to ensure affordability (Pearce et al., 2019). While ACE primarily focuses on evaluating medicine prices, stakeholder involvement remains central to the evaluation process to ensure that outcomes align with the needs of both clinicians and patients (Pearce et al., 2019). Initially, the government will have to set default rules on reimbursement or maximum purchase prices, incentivising the pharmaceutical industry to participate in the agreement and negotiate prices. This will enhance price transparency once a final price is agreed upon. This process serves as a critical criterion for improving the decision-making process, enabling the evaluation of a medicine's actual costs and benefits, which subsequently forms the basis for implementing pricing controls.

### Improve human resources and technical capabilities by effectively disseminating information

Implementing pharmaceutical pricing regulations is anticipated to enhance the collaboration between researchers and policymakers, facilitating the identification of potential challenges and the formulation of effective solutions (Vogler et al., 2015). While Malaysia possesses the human resources to develop policy interventions, it is essential to ensure transparency in information and processes for effectively supporting the fundamental aspect of costing evaluation (O’Donnell et al., 2009). Such transparency is essential, as it enables policymakers to accurately assess the true value of medications prior to the enactment of pricing controls. The value of medicines is evaluated collaboratively with key stakeholders, including the government, patient advocacy groups, academia, and the medical technology industry (O’Donnell et al., 2009).

Information dissemination does not happen naturally and is often passive, leading to inefficiencies in strategic planning (Brownson et al., 2013). Apart from improving stakeholder involvement in the policy evaluation process, it is also essential to discover alternative ways to create long-term changes and knowledge transfer. Key informants have suggested education for the general public to educate consumers on the availability of generic medicines, which could give them more affordable options in line with Babar (2022).

Finally, Chan et al. (2022) suggest that often there are incongruences between trust and mistrust, as individuals have various reasons for deciding whether or not to trust their local authority. This situation may lead to ineffective policy communication, compromising policy delivery and the welfare of the general public. It is essential to recognise that effective communication is bidirectional. Implementing pharmaceutical pricing regulation necessitates multiple rounds of communication (Babar et al., 2024) and public consultations involving key stakeholders, such as pharmaceutical companies, pharmacists, general practitioners, and, most importantly, the general public. At the same time, the policy's beneficiaries, namely the general public, should be able to convey their needs regarding their medical spending to policymakers.

## Conclusion

This study contributes to understanding stakeholders’ views on pharmaceutical pricing regulations. Unlike neighbouring countries such as Thailand and Indonesia, which have implemented pharmaceutical pricing regulations (WHO, 2020), Malaysia is at a watershed moment in considering pharmaceutical pricing regulation as part of its healthcare policy development. Given the current constraints in the public healthcare system, implementing price control measures could ease the government's budgetary burden and potentially improve the general public's access to essential medicines. However, implementing pharmaceutical pricing, such as the ERP policy, has remained a significant challenge as negotiations between key stakeholders have reached an impasse. The lack of price transparency in the pharmaceutical industry poses a challenge for the government in imposing price controls. Therefore, we recommend strengthening requirements for price transparency to facilitate the regulation of pharmaceutical pricing in Malaysia.

## Declaration of generative AI and AI-assisted technologies in the writing process

During the preparation of this work, the authors used generative AI only for grammar checking and to enhance the article's readability. After utilising this tool/service, the authors reviewed and edited the content as needed and took full responsibility for the content of the published article.

## Supplementary Material

Supplementary Material A and B.docx
