# Supplementary material for: Stakeholder engagement and pharmaceutical pricing regulation: a qualitative inquiry
Source: J Pharm Policy Pract. 2025 Sep 5;18(1):2550370. doi: 10.1080/20523211.2025.2550370 (PMC12416026; doi:10.1080/20523211.2025.2550370)
Supplement: Supplementary Material A and B.docx [file JPPP_A_2550370_SM5049.docx]

**Supplementary Material A – Interview Questions**

**Healthcare Professionals^^[[1]](#footnote-1)^^/ NGOs/ Pharmaceutical Reps/ Public Sector**

***Views on The Policy***

1. Do you know how drug are priced in Malaysia?

(Probe: Have you heard of any drug price controls policy or drug regulation? Where did you heard it from?

(Probe: Have you heard of any relevant policies that have been proposed recently?)

**Note:** To test/understand the knowledge of participants on drug pricing in Malaysia.

1. Are you aware that the government has proposed the medicine price control policy in 2019?

(Probe: If **yes**, Can you tell what you understand what has been proposed? Where did you hear this from?)

(Probe: Explain to them what has been proposed in 2019 if their answer is "**don't know**" or "**not sure**".)

In Malaysia, the drug prices in private healthcare currently are not regulated. To address this issue, the government has proposed to impose price control measures for pharmaceutical drugs by adopting external reference pricing, where the price of medicine will be set by calculating a benchmark price by comparing the prices from more than one country. The price controls will allow a regressive markup^^[[2]](#footnote-2)^^ of 10% to 35% on the final products sold by healthcare providers (hospitals, clinics and pharmacies).

**Note:** To understand if they are aware of the proposed drug price controls policy as one of the determinants of interest level for stakeholder analysis.

1. Do you think the current price for medicine are expensive to you? Why? Please elaborate.

(Probe: If **yes**, where do you think it is expensive?

Do you think Malaysia needs a medicine price control? Why or why not?

If **no**, continue on question #4. )

1. Under what circumstances, medicine price control should be applied?

**(**Probe: If price controls were necessary, how would you recommend it would be done? )

(Probe: To understand whether they are supporting medicine price controls.)

1. What would be the biggest challenges faced by Malaysia in implementing a medicine price control?

(Probe: What are the concerns for implementing the policy? In your opinion.)

(Probe: To understand their concerns about the policy.)

1. How would you address these challenges in #5?

(Probe: Who has to be involved?)

(Probe: What solutions/suggestions do you recommend to resolve the problem encountered?)

**Note:** 4-5 is to ask stakeholders hypothetically **if they don't agree** with price controls.

1. In your opinion, how would the implementation of medicine price control affect you (in terms of yourself/ your business/administration)?

(Probe: Do you think implementing price controls on medicine would be an issue for you?)

**Note:** Questions 3 -7 will contribute to the understanding of the barriers/concerns on implementing drug price controls in Malaysia and further develop policy implications.

***Evaluation of Stakeholder Inclusiveness***

1. In your opinion, who are the people/ parties involved in the policy for pharmaceutical pricing? Government, pharmaceutical companies, public health system or the community? Please elaborate. Are there people who should be involved who currently are not?

(Probe: Why? How are they involved?)

**Note:** To map out potential stakeholders involved in the policymaking process.

1. Have you ever been able to communicate your concerns to policymakers? How would you normally do this?

(Probe: If **yes,** what are the outcomes/responses? How many meetings have been held for discussions?

If **not**, what are the main obstacles to reaching out?)

**Note:** To understand stakeholders' influence level (power) and communication.

1. What else would you recommend, other than price controls, to make medicines more affordable and accessible to all?

(Probe: Fact about people unable to access essential medicines.)

**General Public/ Individuals with non-communicable diseases (NCD)**

***General Information***

1. Have you, your family or close friends needed to take medicines for an extended period?

(If **yes**, what type of medicines?)

(If **no,** proceed to question #12)

1. Do you (or your family members/close friends who needed to take medicines for an extended period) have medical insurance coverage?

(Probe: If **yes**, what type of health insurance? Personal insurance, company insurance or others? Please elaborate.)

(Note: For individuals who have NCDs, continue on question #14.

For family members who have NCDs, continue on question #13.)

1. Do you have experience helping your family member/friends with purchasing medicine (that you have mentioned in previous question #12)?

(**Note:** This question only applies to individuals who need to take care of their family members/ friends who have NCDs and are under medication for a longer period.)

If **yes**, continue on question #14.

If **no**, continue on question #15.

**Note:** Question 11-13 is to understand the background of the participants.

***Personal Perceptions***

1. In your opinion, how much the medicine cost could be your major consideration? How much have you spent on the expenses of medication (monthly)?

(**Note:** This question only applies to individuals with NCDs and the general public taking care of their families as identified in #12.)

(Probe: How many per cent (portion) of your income has been spent on buying medicines?)

(Probe: Do you think the current medicine prices are reasonable?)

1. If the medical expenses are too expensive for you, can you speak (or raise your concerns) to someone (for instance, friends, families, community groups or government reps) regarding this issue?

(**Note:** This question only applies to individuals with NCDs and the general public taking care of their families as identified in #13.)

If **yes,** How would you normally do this? What are the outcomes/responses?

**Note:** To understand whether patients can reach out to the stakeholders (as the determinant of power level).

***Views on the policy***

1. In your opinion, who are the people/ parties involved in the policy for pharmaceutical pricing? Government, pharmaceutical companies, public health system or the community? Please elaborate. Are there people who should be involved who currently are not?

(Probe: Why? How are they involved?)

1. Do you think the current price for medicines needs to be changed? Why?
2. Are you aware that the government has proposed the medicine price control mechanism in 2019?

(Probe: Explain to participants what has been proposed in 2019 if the answer is "**don't know**" and "**not sure**".)

In Malaysia, the drug prices in private healthcare currently are not regulated. To address this issue, the government has proposed to impose price control measures for pharmaceutical drugs by adopting external reference pricing, where the price of medicine will be set by calculating a benchmark price by comparing the prices from more than one country. The price controls will allow a regressive markup^^[[3]](#footnote-3)^^ of 10% to 35% on the final products sold by healthcare providers (hospitals, clinics and pharmacies).

(Probe: If **yes**, Can you tell me what you understand about what has been proposed? Where did you hear this from?)

1. In your opinion, how would the implementation of medicine price control affect you (in terms of your daily life)?

(Probe: Do you think implementing price controls on medicine would be an issue for you? What are the concerns for implementing the policy?)

**Note:** Questions 16 -19 will help to understand the barriers/concerns on implementing drug price controls in Malaysia and further develop policy implications.

**Supplementary Materials B – Ethics Approval**

**
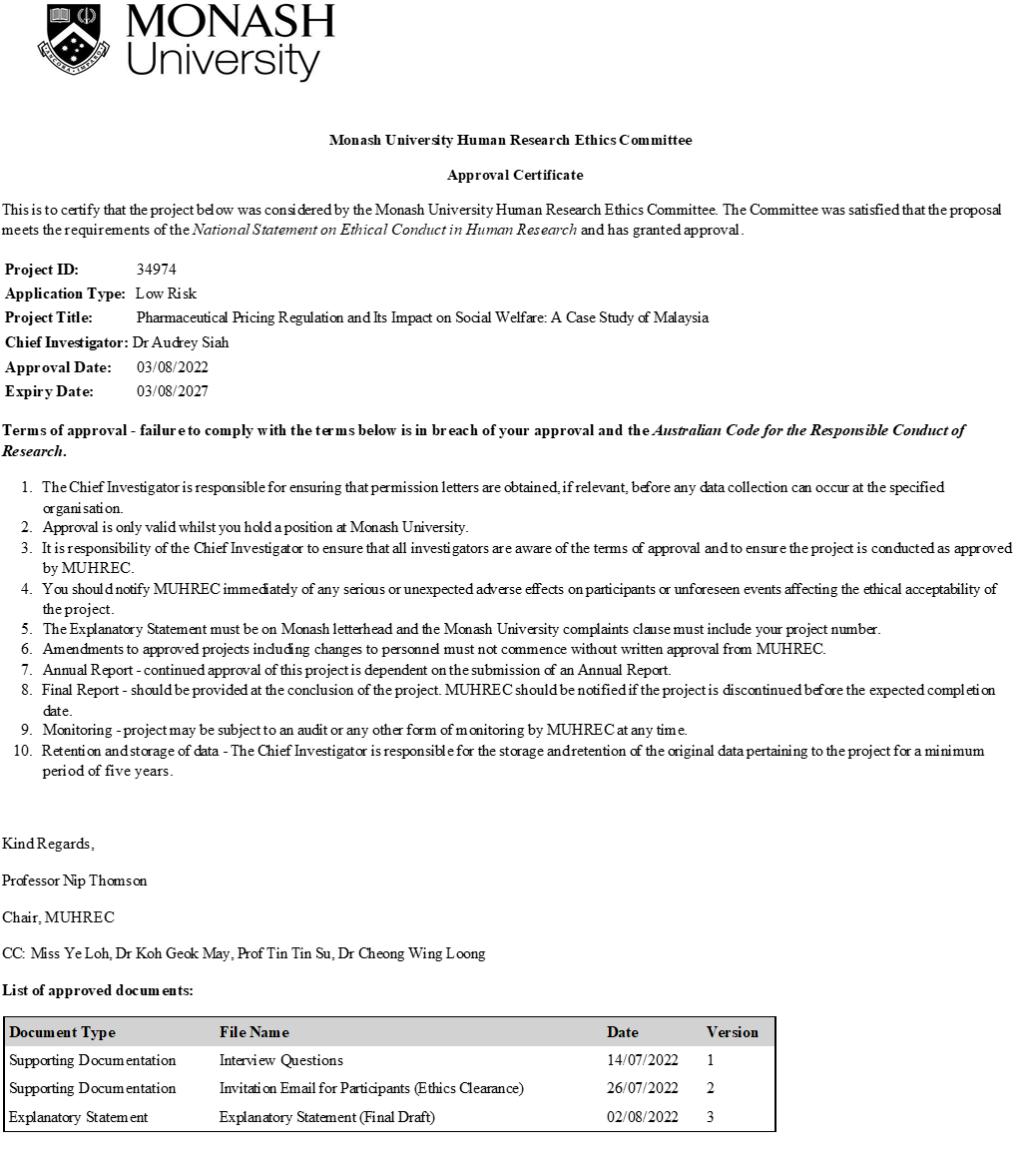
**

1. Pharmacists, GPs, Academicians. [↑](#footnote-ref-1)
2. The mark-up rate decreases as the price of the medicine increases. [↑](#footnote-ref-2)
3. The mark-up rate decreases as the price of the medicine increases. [↑](#footnote-ref-3)
